# Cerebellar Output in Zebrafish: An Analysis of Spatial Patterns and Topography in Eurydendroid Cell Projections

**DOI:** 10.3389/fncir.2013.00053

**Published:** 2013-04-01

**Authors:** Lucy A. Heap, Chi Ching Goh, Karin S. Kassahn, Ethan K. Scott

**Affiliations:** ^1^School of Biomedical Sciences, The University of QueenslandBrisbane, QLD, Australia; ^2^Institute for Molecular Bioscience, The University of QueenslandBrisbane, QLD, Australia; ^3^Queensland Brain Institute, The University of QueenslandBrisbane, QLD, Australia

**Keywords:** zebrafish, cerebellum, eurydendroid, optic tectum, thalamus, topography, Gal4

## Abstract

The cerebellum is a brain region responsible for motor coordination and for refining motor programs. While a great deal is known about the structure and connectivity of the mammalian cerebellum, fundamental questions regarding its function in behavior remain unanswered. Recently, the zebrafish has emerged as a useful model organism for cerebellar studies, owing in part to the similarity in cerebellar circuits between zebrafish and mammals. While the cell types composing their cerebellar cortical circuits are generally conserved with mammals, zebrafish lack deep cerebellar nuclei, and instead a majority of cerebellar output comes from a single type of neuron: the eurydendroid cell. To describe spatial patterns of cerebellar output in zebrafish, we have used genetic techniques to label and trace eurydendroid cells individually and *en masse*. We have found that cerebellar output targets the thalamus and optic tectum, and have confirmed the presence of pre-synaptic terminals from eurydendroid cells in these structures using a synaptically targeted GFP. By observing individual eurydendroid cells, we have shown that different medial-lateral regions of the cerebellum have eurydendroid cells projecting to different targets. Finally, we found topographic organization in the connectivity between the cerebellum and the optic tectum, where more medial eurydendroid cells project to the rostral tectum while lateral cells project to the caudal tectum. These findings indicate that there is spatial logic underpinning cerebellar output in zebrafish with likely implications for cerebellar function.

## Introduction

Coordinated smooth movements and motor learning require the cerebellum, a structure located in the hindbrain of all vertebrates (Glickstein et al., 2011). Cerebellar processing is believed to be based on comparisons between the intended outcomes of motor programs and sensory information reflecting the actual outcomes (Miall et al., 1993; Blakemore et al., 1998; Tseng et al., 2007). Mismatches are indicative of failures of motor programs, and the nature of the mismatches provide information with which the cerebellum can calibrate the associated motor programs (Miall et al., 1993; Tseng et al., 2007).

The cerebellar cortex is composed of a highly ordered, repeating structure made up of the granule cell, Purkinje cell (PC), and molecular layers (Dow and Moruzzi, 1958). PCs are the points of convergence for two pathways of information: the climbing fibers from the inferior olive, and the mossy fibers principally from the pontine nuclei (via granule cell parallel fibers). As such, they are believed to have a role in identifying discrepancies between intended and actual outcomes from motor programs (Albus, 1971). The PCs send their inhibitory signals out of the cerebellar cortex and into the deep cerebellar nuclei (DCN), which provide cerebellar output (Eccles, 1971). The DCN are also a point of convergence for the climbing and mossy fibers, and may themselves have important roles in detecting motor errors (Miles and Lisberger, 1981). Indeed, the relative importance of plasticity in PCs versus DCN plasticity is still the topic of intense debate (reviewed by Carey, 2011).

Anatomically, DCN outputs have been shown to send both excitatory and inhibitory information to areas involved in sensory integration such as the superior colliculus, sensory nuclei of the thalamus (Andrezik et al., 1984; Aumann et al., 1994; Sultan et al., 2012), and motor areas such as the inferior olive and premotor regions of the thalamus (Andrezik et al., 1984). The patterns of output activity generated by the DCN have been studied electrophysiologically in both primates and rodents (Thach, 1968; Hepp et al., 1982; Hoebeek et al., 2010), but the ways in which these patterns subserve motor learning are less well understood. These limitations are partially due to the complexity of the DCN themselves. As a result, research in a simpler and more experimentally accessible model may be beneficial for describing cerebellar output and the ways in which the output guides motor learning.

Zebrafish are proving to be a particularly advantageous model system for behavioral and functional circuit analysis, largely owing to their optical transparency and external development (Scott and Baier, 2009; Friedrich et al., 2010; Simmich et al., 2012). These characteristics, in combination with the quickly developing field of optogenetics, permit the observation and manipulation of neural activity *in vivo*, and may therefore aid in describing patterns of cerebellar activity and output that subserve motor learning. Therefore, it is important to develop a detailed and comprehensive anatomical description of cerebellar circuits in zebrafish that can serve as a scaffold for future functional mapping. The zebrafish cerebellum is composed of three lobes, which are, from rostral to caudal, the valvula cerebelli, corpus cerebelli, and vestibulolateral lobe (Finger, 1983; Bae et al., 2009). As in the mammalian cerebellum, the valvula and corpus cerebelli contain the granule cell, PC, and molecular layers. Reports differ on the structures of layers within the vestibulolateral lobe, which may vary among teleost species (Bass, 1982; Murakami and Morita, 1987; Zhang et al., 2008; Bae et al., 2009). Development of the zebrafish cerebellum starts at 2 days post fertilization (dpf), with complete and functioning circuits by 6 dpf (Aizenberg and Schuman, 2011; Hibi and Shimizu, 2012). As in mammals, the glutamatergic neurons (granule and eurydendroid cells) are derived from the upper rhombic lip while GABAergic neurons (Golgi and PCs) come from the ventricular zone (Kani et al., 2010).

Although there are broad similarities in the structure, connectivity, and development of the zebrafish and mammalian cerebella, a significant difference lies in their output structures. In contrast to mammals, teleosts do not have DCN that are spatially segregated from the cerebellar cortex. Instead, they have a single type of neuron within the cerebellar cortex, the eurydendroid cell, that provides cerebellar output (Finger, 1983; McFarland et al., 2008; Bae et al., 2009). These cells are post-synaptic to PCs, receive input from parallel fibers and possibly climbing fibers, and extend axons beyond the cerebellum (Bae et al., 2009; Hibi and Shimizu, 2012), indicating that they occupy the same circuit position as do the DCN in mammals. Previously, multiple subtypes of eurydendroid cells have been described in both scorpion fish and goldfish, differing in appearance, distribution, and target structures (Murakami and Morita, 1987). These descriptions have shown that type A eurydendroid cells are in the caudal lobe of the cerebellum and project largely to the oculomotor complex, whereas type B eurydendroid cells are located in the valvula and corpus cerebelli and project to a much broader range of structures, including the brainstem, the nucleus ventromedialis thalami, and the nucleus ruber (Murakami and Morita, 1987). Dye-tracing experiments in goldfish have shown that eurydendroid cell subtypes with different morphologies target specific regions including the optic tectum, thalamus (Ikenaga et al., 2006), inferior olive, and hindbrain reticular formation (Finger, 1983). In mormyrid fish, different lobes of the corpus cerebelli target different structures. The first lobe of the corpus cerebelli targets structures including the nucleus of the fasciculus longitudinalis medialis, the trigeminal motor nucleus, and the tectum whereas the third lobe targets the midbrain tegmentum, the torus longitudinalis, and the nucleus reticularis superior (Meek et al., 1986a,b).

It is not clear, however, whether there are multiple eurydendroid cell subtypes in larval zebrafish, whether different parts of the zebrafish cerebellum target different brain regions, or whether topography exists between the cerebellum and its targets in teleosts.

Transgenesis, the process of delivering exogenous genes into a model system’s genome, is an effective approach for labeling neural structures for anatomical description (Feng et al., 2000; Kawakami, 2004; reviewed by Luo et al., 2008). The Gal4-UAS system (Brand and Perrimon, 1993; Scheer and Campos-Ortega, 1999), which allows separate control over the location of expression and the marker being expressed, has brought particular utility to the labeling of circuits for anatomical analysis (Scott, 2009). This has been especially true in the zebrafish model system, since fluorescently labeled neurons can be imaged in live, intact animals. As a result of several enhancer trap screens using Gal4 (Davison et al., 2007; Scott et al., 2007; Asakawa et al., 2008; Distel et al., 2009; Scott and Baier, 2009), hundreds of lines of zebrafish exist with Gal4 expression in specific parts of the nervous system.

While these Gal4 lines exhibit their overall expression patterns when crossed to any line carrying a UAS:fluorophore transgene, this overall expression is often insufficient for judging the connectivity or cellular composition of the population of Gal4-positive cells. To improve upon this, past studies have expressed fluorophores that are targeted specifically to pre- or post-synaptic terminals, thus revealing the neurons’ dendrites or axonal terminals specifically (Niell et al., 2004; Meyer and Smith, 2006). The individual neurons composing an expression pattern can be visualized by driving highly variegated expression, either through injection of a plasmid containing the gene for a UAS-linked fluorophore (Scheer and Campos-Ortega, 1999), or through the use of a highly variegated UAS:GFP (Xiao et al., 2005; Scott et al., 2007; Scott and Baier, 2009; Wyart et al., 2009; Simpson et al., 2013). Since these approaches allow for single neurons to be seen in the context of the expression pattern as a whole, they provide a powerful tool for generating a catalog of a brain region’s cell types, each in anatomical detail and spatial registration.

In this study, we describe a Gal4 ET line (*Gal4^s1168t^*) with expression in the cerebellum, and we use this line to map the projections of eurydendroid cells in larval zebrafish. Using overall expression, a pre-synaptic marker, and imaging of individual eurydendroid cells, we describe the anatomy of cerebellar projections to the tectum and thalamus, thus revealing the spatial and topographic properties of these projections.

## Materials and Methods

### Generation of animals

All procedures were performed with approval from the University of Queensland Animal Ethics Committee (in accordance with approvals QBI/811/07 and SBMS/362/10/NHMRC). Adult fish were maintained, fed, and mated as previously described (Westerfield, 2000). The wild-type strain Tupfel long fin (TL) and the pigment free *nacre* mutant (Lister et al., 1999) were used throughout the experiments. The transgenic lines *Gal4^s1168t^* (Scott and Baier, 2009) *pou4f3: GAL4, UAS:GAP-GFP* (*BGUG*) (Scott et al., 2007), and *UAS:Kaede* (Scott et al., 2007) have previously been described. To make the *UAS:mCherry* line, monomeric Cherry with a K-ras membrane localization signal was subcloned into the pT2KXIGΔ in vector (Kotani et al., 2006) using *Eco*RI and *Not*I, downstream of a 14X UAS element. Similarly, the *UAS:synaptophysin-GFP* (*UAS:syn-GFP*) construct was made by subcloning *synaptophysin-GFP* (Meyer and Smith, 2006) into the pT2KXIGΔ in vector (Kotani et al., 2006) using *Eco*RI and *Not*I, downstream of a 14X UAS element. Embryos were injected at single cell stage with a solution containing 25 ng/μL plasmid DNA, 50 ng/μL transposase mRNA, and 0.04% Phenol Red.

To create variegated expression of a plasmid, eggs were injected with a *UAS:Brainbow2.1* construct (Livet et al., 2007) flanked by Tol2 transposon elements (Kawakami, 2004). This construct was generated by inserting *Brainbow2.1*, in reversed orientation, into the pME-MCS vector (Kwan et al., 2007) using *Xho*I and *Xba*I. *UAS:Brainbow2.1* was then generated in the Tol2kit (Kwan et al., 2007). Injection mix consisted of 100 ng/μL transposase, 75 ng/μL *UAS:Brainbow2.1*, and 50 ng/μL *Cre* recombinase, diluted in water, and phenol red was added to allow visualization during injections. *Gal4^s1168t^; UAS:mCherry* embryos were collected within 20 min of fertilization, and were injected under a dissecting light microscope at the single or two cell stage. At 24 and 48 h post fertilization, embryos were sorted for transient Brainbow expression, indicated by yellow and green fluorescent protein (YFP and GFP), and YFP-positive cells in these animals were imaged at 6 dpf. Images from a total of 21 larvae generated the data for the single cell analyses in this study, with an additional 9 larvae providing data from pairs or small clusters of cells.

### Identification of genomic insertion sequences

The insertion site for *Gal4^s1168t^* was mapped as described by Kotani et al. (2006) and Laplante et al. (2006), using *Mbol* and linker mediated PCR. The following modifications were made to the primer sequences used by Kotani et al. (2006): Ap1: 5′GGATTTGCTGGTGCAGTACAG3′, Ap2: 5′AGTACAGGCCTTAAGAGGGA3′, L100-Out: 5′AGATTCTAGCCAGATACT3′, R100-Out: 5′GTATTGATTTTTAATTGTA3′, L150-Out: 5′GAGTAAAAAGTACTTTTTTTTCT3′, R150-Out: 5′TAATACTCAAGTACAATTTTA3′, L175-Out: 5′CTTTTTGACTGTAAATAAAATTG3′, R175-Out: 5′TCTTTCTTGCTTTTACTTTTACTTC3′.

### Mounting and microscopy

At 1 dpf, 25 μL of 7.5% phenylthiourea (PTU) in solution with dimethyl sulfoxide was added to 100 ml E3 media. This media was used to suppress the formation of skin pigmentation that interferes with imaging. Embryos were observed for fluorescence at 48 h post fertilization, and imaging was carried out at 6 or 7 dpf. Larvae with the genotype *Gal4^s1168t^; UAS:mCherry, UAS:Brainbow* or *Gal4^s1168t^; UAS:mCherry, BGUG* were mounted dorsal side up in 2% low melt agarose (Progen Biosciences, Murarrie, QLD, Australia). In some cases, photoconverted red Kaede was used in the place of mCherry as described (Scott et al., 2007). Imaging was carried out on the Zeiss-LSM 510 upright confocal microscope using a 543 nm laser and 560 nm long pass filter for mCherry and 488 nm laser and 505–530 nm band pass filter for YFP and GFP. Images were taken using 10, 20, and 63× objectives.

### Image analysis

Images were viewed on ImageJ version 1.45s (U.S. National Institutes of Health, Bethesda, MD, USA), and the plugin “Neurite tracer” (Longair et al., 2011) was used to trace axon projections from the cell body to termination. The medial-lateral, rostral-caudal, and dorsal-ventral positions of the cell body within the cerebellum were measured, and were converted to percentage values.

Separate red and green channels were used on Imaris version 7.4 (Bitplane, Zürich, Switzerland) to create three-dimensional tracings of individual cells. Cells imaged over multiple stacks were stitched together using the freeware XUV stitch program (Emmenlauer et al., 2009). Using the Neurite Wizard function on Imaris version 7, the cell body and axon termination of each neuron were identified and joined using the manual trace function.

To determine the coordinates of a cell termination in the tectal neuropil, the three-dimensional Imaris image was rotated so that the rostral-caudal axis of the tectum was vertical in the viewing panel.

### Statistical analysis

The position of the cell body in the cerebellum and its termination in the tectal neuropil were compared against each other in all axes to see whether correlations were present, using Graph Pad Prism version 6 for Windows (GraphPad Software Inc., La Jolla, CA, USA) and R freeware (R Core Team, Vienna, Austria, http://www.R-project.org). A Schapiro-Wilk test for normality was performed, and all datasets across tectal and cerebellar axes were found to be normally distributed. A Pearson’s correlation test was used to test for correlations within the data. A Holm test was used to adjust *p* values for multiple comparisons. Significance was accepted as *p* ≤ 0.05.

## Results

### The *Gal4^s1168t^* insertion is located between two genes with cerebellar expression

The transgenic zebrafish line *Gal4^s1168t^*, with Gal4 expression in the cerebellum and trunk muscles, was identified in an enhancer trap screen performed by Scott et al. (2007), and further characterized by Kani et al. (2010) as having expression in atoh1a-positive cells within the cerebellum. The insertion site of *Gal4* in this line is in an intergenic region on chromosome nine, 7.8 kb downstream of the *insig2* gene and 11.8 kb upstream of *eng1a*. These genes are homologous, respectively, to the genes Insig2 and EN1 in humans and mice, and the expression levels in multiple mammalian tissues have been identified. The gene Insig2 is highly expressed in the human nervous system, and expression is highest in the cerebellum and thalamus (Becanovic et al., 2010), as well as skeletal muscle (Thierry-Mieg and Thierry-Mieg, 2006). In mouse, high expression of EN1 has been shown in the cerebellum (Donarum et al., 2006), superior colliculus, and brain stem (Zapala et al., 2005). Given the highly overlapping expression patterns of these two genes, it is difficult to judge whether the expression of Gal4 in the cerebellum and trunk muscles of *Gal4^s1168t^* larvae results from enhancers belonging to one, the other, or both genes.

### Cerebellar expression in the *Gal4^s1168t^* enhancer trap line

**Figure 1:**
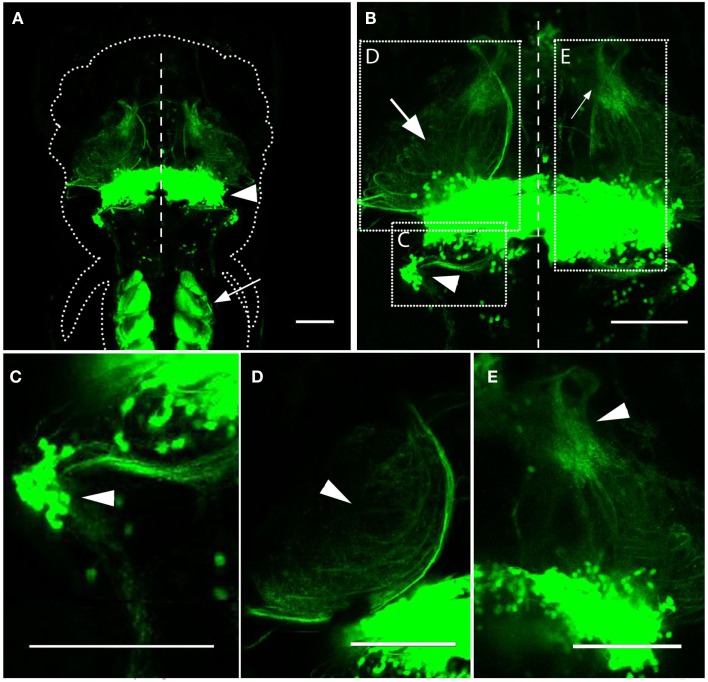
**Expression in the *Gal4^s1168t^* line**. **(A)** Dorsal image of a 6 dpf *Gal4^s1168t^; UAS: Kaede* larva, anterior at the top. The approximate boundary of the animal is shown with a dotted line, and the midline of the animal is indicated by a dashed line. Expression is strongest in the cerebellum (arrowhead) and trunk muscles (arrow). **(B)** A higher-magnification image of the same animal in **(A)**. Neurites between the cerebellum and the CC (arrowhead), tectum (arrow), and thalamus (small arrow) are evident. The regions shown in **(C–E)** are indicated with dashed boxes, and the midline is depicted by a dashed line. **(C)** Neurites are evident between the cerebellum and the CC (arrowhead), where some Gal4-positive cell bodies also reside. **(D)** Cerebellar neurites also project to the tectal neuropil (arrowhead). **(E)** More sparse neurites from the cerebellum are found in the thalamus (arrowhead). Scale bars represent 100 μm. A *z*-series of **(B)** can be viewed in Movie S1 in Supplementary Material.

Our initial characterization of the *Gal4^s1168t^* line involved imaging *Gal4^s1168t^; UAS:Kaede* larvae at 6 dpf. Observations of these animals were in keeping with preliminary descriptions for this line from the original screen (Scott and Baier, 2009). We found expression to be strongest in trunk muscles and in the cerebellum (Figure 1A), and noted the presence of neurites exiting the cerebellum (Figures 1B–E). Structures sharing projections with the cerebellum included the crista cerebellaris (CC) in the hindbrain (Figure 1C), the optic tectum in the midbrain (Figure 1D), and the thalamus in the forebrain (Figure 1E). Few neurons outside of the cerebellum were seen to be Gal4-positive. Movie S1 in Supplementary Material shows a *z*-series through the structures shown in Figure 1B.

A majority of these neurites exit the cerebellum and proceed toward the optic tectum and thalamus. These include two groups of neurites, with the first exiting the ventral lateral part of the corpus cerebelli, extending ventrally and rostrally, and then traveling dorsally to pass through the deep layers of the tectal neuropil (Figure 1B; Movie S1 in Supplementary Material). The second group includes neurites that extend rostrally from the ventral cerebellum throughout its medial-lateral range, and project more directly into the deep tectal neuropil (Movie S1 in Supplementary Material). Combined, these neurites blanket the deepest layers of the tectal neuropil, making it impossible to determine the structures of the individual neurites. A subset of the neurites in the tectal neuropil extend further into the dorsal thalamus, but viewing these neurites *en masse*, we are not able to judge whether they have any other distinguishing characteristics that could be used to classify them as a subtype of cerebellar projection neuron. We also observed axon tracts exiting the cerebellum to other regions of the midbrain and forebrain (Movie S1 in Supplementary Material), but these were inconsistent, and were not confirmed in the more detailed analyses presented below.

Axons from the caudal cerebellum were seen targeting the CC (Figure 1C). These axons traveled laterally to reach the CC at depths of between 10 and 30 μm below the dorsal surface of the animal. Since Gal4-positive cell bodies are present in the CC (Figure 1C), the possibility also exists that these projections run in the opposite direction, from the CC to the cerebellum. That said, axon tracts from Purkinje and granule cells originating in the valvula cerebellum have been reported to project to the CC (Miyamura and Nakayasu, 2001). Therefore axons traveling between the CC and the cerebellum are likely to have originated from cells in this area.

**Figure 2:**
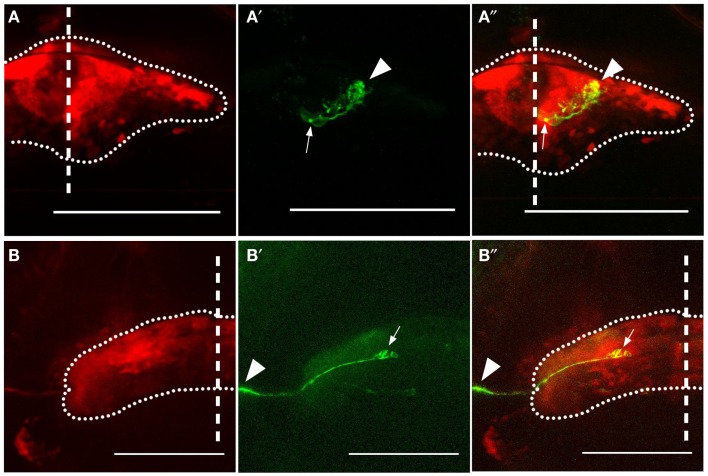
**Expression in Purkinje and eurydendroid cells**. Expression of GFP in a Purkinje cell **(A–A**″**)** and eurydendroid cell **(B–B**″**)** in *Gal4^s1168t^; UAS:Kaede; BGUG* transgenic larvae are shown. For each cell, the overall expression pattern is visible as red Kaede **(A,B)**, and the approximate bounds of the cerebellum are indicated with a dotted line. The Purkinje cell has its soma [arrow in **(A**′**,A**″**)**] located ventrally in the cerebellum, with dendrites (arrowhead) elaborating in the dorsal region, presumed to be the molecular layer. The eurydendroid cell [arrow in **(B**′**,B**″**)**] extends a neurite laterally and beyond the cerebellum (arrowheads). This neurite is temporarily lost from the image as it plunges ventrally before resurfacing. Scale bars represent 100 μm, and the midline is indicated with a vertical dashed line.

In order to identify the Gal4-positive cells within the cerebellum, we observed variegated expression of GFP provided by the *BGUG* transgenic line (Scott et al., 2007). For reasons that are unclear, expression from *UAS:GFP* in this construct is extremely sparse, and this permits GFP-positive neurons to be visualized individually (Scott, 2009). We found two cell types that were regularly labeled using this technique in the *Gal4^s1168t^* line: PCs (Figures 2A–A″) and eurydendroid cells (Figures 2B–B″). The latter have previously been described as the output neuron of the teleost cerebellum (Finger, 1983; Meek et al., 1986a,b; Murakami and Morita, 1987; Ikenaga et al., 2006; Bae et al., 2009; Kani et al., 2010), and can be seen contributing projections beyond the cerebellum in our *BGUG* analyses (arrowheads, Figures 2B′,B″). From this, we suggest that the neurites observed in this ET line are efferent axons from cerebellar eurydendroid cells.

### Brain structures targeted by cerebellar output

**Figure 3:**
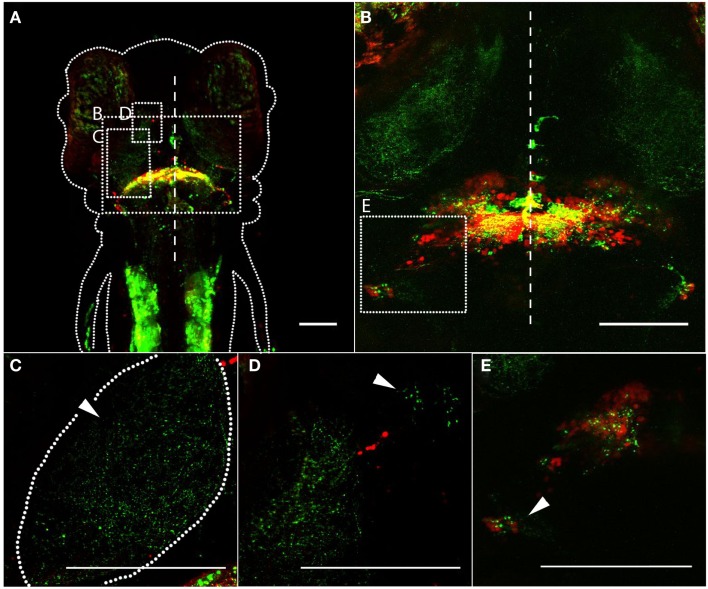
**Synaptic targets of the cerebellum, revealed by a pre-synaptic marker**. In *Gal4^s1168t^; UAS; Kaede; UAS:syn-GFP* transgenic larvae, photoconverted Kaede is shown in red, and syn-GFP is shown in green. **(A)** Dorsal image of a 6 dpf larva, anterior at the top. The approximate boundary of the animal is depicted by a dotted line, and the midline of the animal is shown by the dashed line. The approximate locations of **(B–D)** are shown with dashed boxes. **(B)** A higher-magnification dorsal image of a 6 dpf larva, with the midline indicated by a dashed line. The greatest concentration of pre-synaptic terminals is in the cerebellum itself, but regions outside of the cerebellum **(C–E)** also show GFP. The approximate location shown in **(E)** is indicated with a dashed box. **(C)** Cerebellar synaptic output in the tectal neuropil (arrowhead) is shown. The approximate boundaries of the neuropil are indicated with a dashed line. **(D)** A high-magnification image showing cerebellar synaptic output in the thalamus. The presence of a smaller cluster of GFP puncta (arrowhead) indicates that multiple distinct parts of the thalamus may be targeted. **(E)** Synapses are apparent in the CC, shown with an arrowhead. Scale bars represent 100 μm. A *z*-series of **(B)** can be viewed in Movie S2 in Supplementary Material.

We next crossed *Gal4^s1168t^* to a transgenic line for *UAS:syn-GFP*, leading to the expression of GFP at axonal terminals (Meyer and Smith, 2006; Li et al., 2010). This was intended to confirm that the neurites that we have observed are, in fact, output axons, and to judge how well the above-described cerebellar projections register against this more direct readout of output. *Gal4^s1168t^; UAS:Kaede; UAS:syn-GFP* larvae (Figure 3A), with photoconverted red Kaede (Ando et al., 2002) predominantly in cell bodies and GFP in axonal terminals, show cerebellar output to the deep tectal neuropil (Figure 3C), the thalamus (Figure 3D), and the CC (Figure 3E). In the case of the CC, we also observe cell bodies (red, Figure 3E), raising the possibility that the syn-GFP signal in the CC is actually due to local circuitry, rather than cerebellar output. In addition to this remote labeling, there are robust GFP signals within the cerebellum itself (Figures 3A,B), likely belonging to the axons of PCs, and in the trunk muscles (Figure 3A).

This corresponds well to the above description of the tracts exiting the cerebellum in this line. It confirms that the tracts comprise axons, and that the axons form synapses in the structures in which they terminate or pass through. This means, for example, that the tectum receives output from eurydendroid cells, rather than simply being a conduit for axons terminating in the thalamus. It is not clear from this analysis, however, whether there are distinct populations of eurydendroid cells targeting the tectum, the thalamus, or both.

### Spatial mapping of individual eurydendroid cells

To resolve ambiguities like those just described, and to determine whether there is topographic organization of cerebellar outputs, we next undertook a systematic description of eurydendroid cells in terms of their cell body positions, target areas, and points of axon termination. We employed two methods: sparse expression from the *BGUG* transgene (Scott et al., 2007; Scott and Baier, 2009) and variegated expression resulting from injections of *UAS:Brainbow* DNA (Livet et al., 2007). In both cases, rates of labeling were low. In the case of *Gal4^s1168t^; UAS:mCherry, BGUG* and *Gal4^s1168t^; UAS:Kaede, BGUG* triple transgenic larvae, a small percentage (approximately 0.4%) expressed mGFP in resolvable cells within the cerebellum (*n* = 9 larvae, each with an individually resolvable eurydendroid cell). *UAS:Brainbow* injections into *Gal4^s1168t^; UAS:mCherry* embryos resulted in single cell labeling within the cerebellum 1.9% of the time (*n* = 12, with 16 resolvable cells). In a few cases, two or more cells were labeled in the same animal, but could be unambiguously traced as individuals.

The optic tectum was seen to receive output from the cerebellum at a single cell level (*n* = 21 animals), and several pairs or small clusters of eurydendroid cells were seen targeting the dorsal thalamus (*n* = 9 animals). Although they appear to receive axon tracts from the cerebellum, the CC and hindbrain were not targeted by the individual cells that we observed. This could be a function of relatively sparse innervation to these structures combined with a limited number of observed cells, or could be due to possible unintended biases in the labeling methods.

### Subtypes of eurydendroid cells targeting the tectum and thalamus

To determine whether spatial organization exists in cerebellar output, we compared the locations of eurydendroid cells’ somata with the termination points of their axons. Cell body locations were quantified as a percentage value within a given axis of the cerebellum. We found eurydendroid cells to be distributed broadly throughout the cerebellum, from 7 to 97% along the medial-lateral axis, 7–95% of the rostral-caudal axis, and 21–83% of the dorsal-ventral axis.

**Figure 4:**
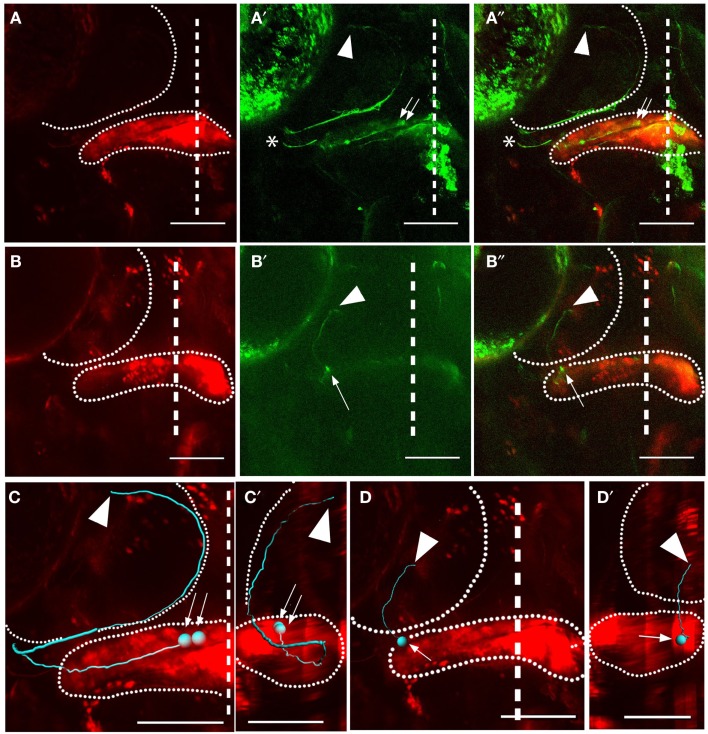
**Subtypes of eurydendroid cells**. Red shows Kaede and green shows GFP (expressed from the *BGUG* transgene). **(A)** A dorsal *z*-projection of a pair of cells from the medial cerebellum targeting the thalamus. **(A)** Shows the red channel (photoconverted Kaede), and **(A**′**)** shows the green channel (GFP), with an arrow indicating the cell bodies and arrowhead indicating the corresponding termination points. **(A**″**)** Shows a merged image of **(A,A**′**)**. Imaging of this projection is weak when the neurites are in deep ventral positions, and overlying neurites from other neurons [asterisks in **(A**′**,A**″**)**] can obscure these *z*-projections, so these cells are best viewed as *z*-series (Movies S3 and S4 in Supplementary Material). **(B)** A dorsal *z*-projection of a cell from the intermediate cerebellum targeting the tectum. The positions of the cell body (arrow) and axon terminal (arrowhead) are indicated. **(C,D)** show dorsal Imaris tracings of cells shown in **(A**′**,A**″**)** and **(B**′**,B**″**)**, respectively, with cell bodies indicated by arrows and terminations indicated with arrowheads. Both the cerebellum and tectum are indicated with dotted lines. **(C**′**–D**′**)** show sagittal views of tracings in **(A**′**,A**″**)** and **(B**′**,B**″**)**, dorsal to the left, with the cerebellum and tectum indicated with dotted lines. The cell in **(A**′**,A**″**)** can be seen to extend to deep ventral positions before extending to the tectum and thalamus, while cell in **(B**′**,B**″**)** has a relatively flat sagittal profile. Scale bars represent 100 μm. For *z*-series of the cells shown in **(A,B)**, see Movies S3 and S4 in Supplementary Material, where the paths of the axons are clearer than in the two-dimensional shown here.

We observed cerebellar output to the thalamus from eurydendroid cells whose cell bodies were in the most medial part of the cerebellum (approximately the most medial 20% of the medial-lateral axis) (Figures 4A–A″). These cells typically appeared in pairs (see Figure 4) or small clusters (*n* = 9 animals). The axons of these medial cells exit the lateral corpus cerebelli, requiring them to project laterally through the cerebellum before exiting (Figures 4A″,C,C′). After exiting the cerebellum, their axons project ventrolaterally before turning dorsally to the medial edge of the tectal neuropil. They then follow the medial/ventral edge of the tectal neuropil before exiting the rostral tectum, and projecting ventrally to the thalamus. These cells were often seen extending small neurites (<10 μm) radially into the tectal neuropil as they passed through. Although it is not proven by these observations, these seem likely points for *en passant* synapses in the deep tectal neuropil.

We observed output to the optic tectum, without further extensions to the thalamus, in 25 individually labeled eurydendroid cells (*n* = 21 larvae). These cells (Figures 4B–B″,D,D′) were located throughout most of the cerebellum’s medial-lateral axis (roughly the lateral 80%). Axons from these cells project ventrally within the cerebellum before exiting at ventral-rostral points along the cerebellum’s medial-lateral axis. They then enter the tectal neuropil on its ventral side, and travel dorsally until reaching their termination points within the deep layers of the tectal neuropil. In contrast to the medial eurydendroid cells that project to the tectum and thalamus, these more numerous eurydendroid cells do not form thick fascicles, and rather extend their axons individually to the tectum.

In addition to these two types of eurydendroid cell, we observed one example of a projection neuron with its cell body in the tegmentum, adjacent to the cerebellum, and with long projections to the hypothalamus (not shown). This type of neuron, which is not a eurydendroid cell, has previously been described in the *Gal4^s1168t^* line by Kani et al. (2010).

### Topography in cerebello-tectal projections

**Figure 5:**
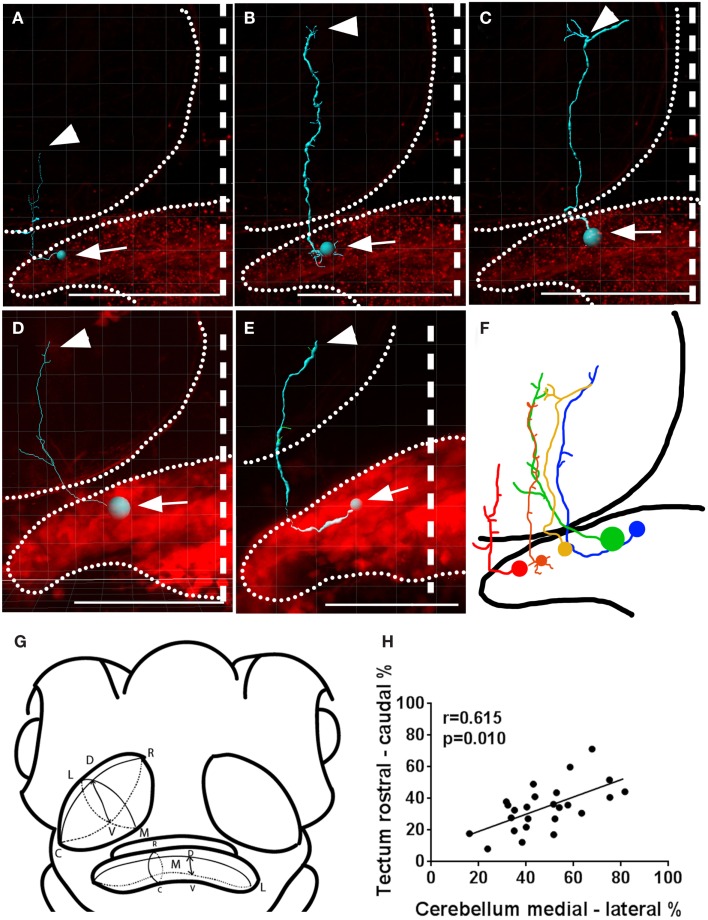
**Topography between the cerebellar medial-lateral and the tectal rostral-caudal axes**. **(A–E)** Imaris tracings of individual eurydendroid cells with the cell bodies (arrows) and axonal terminals (arrowheads) indicated. The expression pattern as a whole is shown in red and the boundaries of the tectal neuropil and cerebellum are indicated with dotted lines. **(F)** A representation of cells in **(A–E)**, registered against one another spatially, both in the cerebellum and the tectal neuropil. **(G)** Shows the orientations in which the axes were measured in a way that compensates for the tectal neuropil’s slanted orientation (R, rostral; C, caudal; M, medial; L, lateral; D, dorsal; V, ventral). **(H)** A correlation was found between the cell body position within the medial-lateral axis of the cerebellum and the rostral-caudal position of termination points within the tectal neuropil, as shown by a Pearson’s product moment correlation, *r* = 0.645, *p* = 0.010. Larvae are 6 dpf with genotypes *Gal4^s1168t^; UAS:mCherry, UAS:Brainbow* **(A–C)** or *Gal4^s1168t^; UAS:Kaede:UAS; BGUG* **(D,E)**. Scale bars indicate 100 μm, and the midline is shown with a dashed line.

Since the optic tectum is a topographically organized structure (Sajovic and Levinthal, 1982; Collin and Pettigrew, 1988; Stuermer, 1988), we investigated whether topography exists between the cerebellum and the tectum. We did this by comparing the position of individual eurydendroid cells’ bodies in the cerebellum with the termination points of their axons within the tectal neuropil (Figure 5). We found significant topography between the cell body position along the medial-lateral axis of the cerebellum and the axon termination point along the rostral-caudal axis of the tectal neuropil (Pearson’s correlation, *r* = 0.615, *p* = 0.010). Among the eurydendroid cells projecting exclusively to the tectum, the more medial ones project to the rostral parts of the optic tectum, and the more lateral ones project to caudal areas of the tectal neuropil (25 individual cells, *n* = 21 larvae, regression shown in Figure 5H). This topography was overlaid on a trend toward axon terminations in the rostral tectal neuropil, as a majority of all eurydendroid cells observed extended into the rostral half of the tectum (Figure 5H).

**Figure 6:**
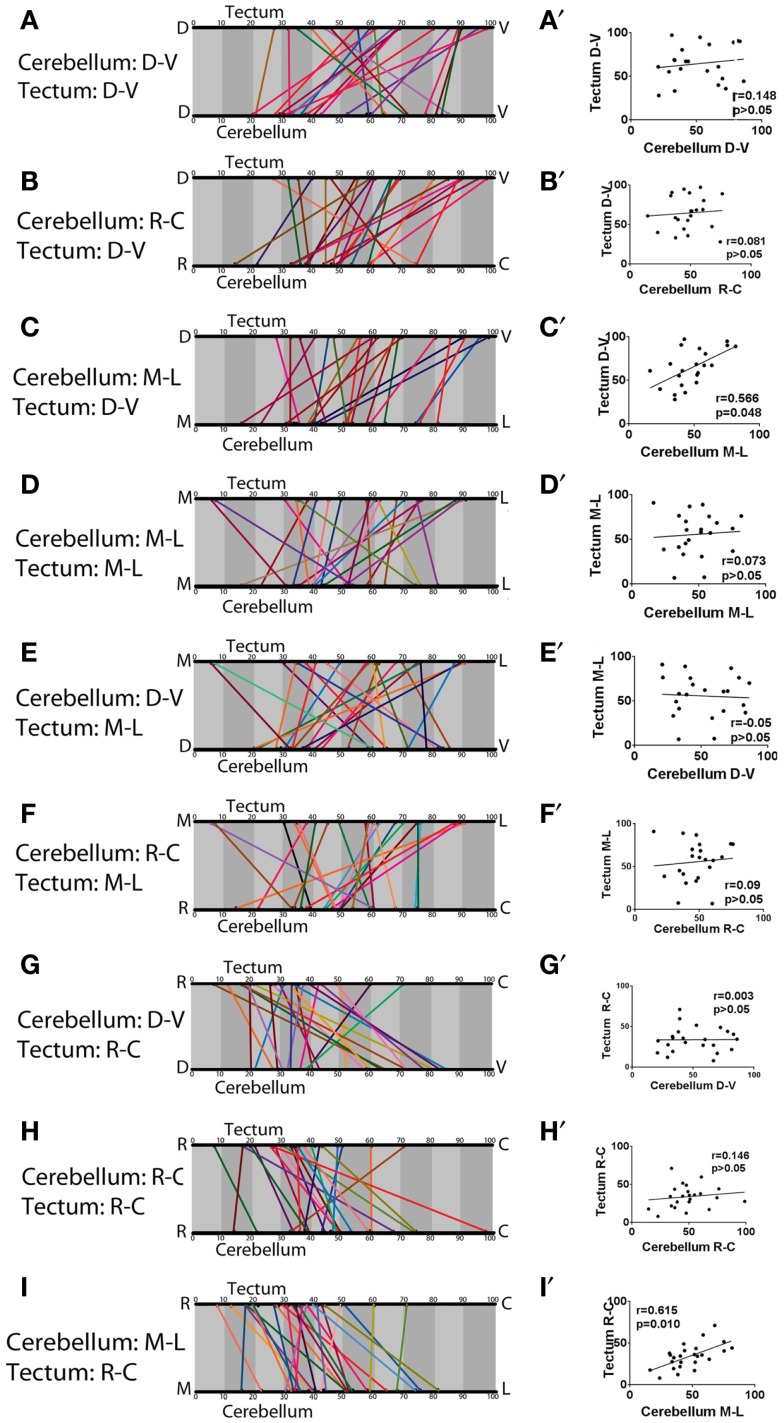
**A test for topography across all cerebellar and tectal axes**. All pairs of axes between the cerebellum and tectum were compared to determine whether topography exists. The majority of comparisons showed no significant correlation. The exceptions are between the medial-lateral axis of the cerebellum and the dorsal-ventral axis of the tectum **(C)** (Pearson’s *r* coefficient = 0.566, *p* = 0.048), and the medial-lateral axis of the cerebellum and rostral-caudal axis of the optic tectum **(I)**, also shown in Figure 5. (Pearson’s *r* = 0.615, *p* = 0.010). **(A–I)** show linear representations of the cell body position and corresponding axon terminal position of each cell, represented as percentages along the indicated axes. **(A**′**–I**′**)** show scatter plots with each eurydendroid cell as a single point and a line of best fit.

We continued this analysis to look for trends between all pairs of axes in the cerebellum and tectum, and (with the exception of the correlation described above) these showed little or no evidence for further topography (Figure 6). It should be noted that a significant correlation was found to exist between the medial-lateral axis of the cerebellum and the dorsal-ventral axis of the optic tectum (Figure 6C) (Pearson’s correlation, *r* = 0.566, *p* = 0.048). This, however, is likely a combined product of our measurement technique and the tectal neuropil’s lens-shaped structure, rather than a biologically interesting pattern of connectivity. Since the tectal neuropil is thinner at its edges than its center, and since a majority of eurydendroid terminals are in the rostral neuropil, our more rostral terminals would show a higher percentage value on the dorsal-ventral axis, even if they are a set distance from the floor of the neuropil. Given the topography between the medial-lateral axis of the cerebellum and the rostral-caudal axis of the tectum, this introduces a bias that makes the medial eurydendroid cells, which terminate rostrally, appear to be in more dorsal layers of the neuropil. Direct measurements of the terminals’ distances from the floor of the neuropil do not show a significant correlation with corresponding cell body positions in the medial-lateral axis of the cerebellum (Pearson’s correlation, *r* = −0.467, *p* = 0.263), indicating that there is no important targeting between spatially distinct eurydendroid cells and different dorsal-ventral layers of the tectal neuropil.

## Discussion

### Targets and spatial organization of cerebellar output

**Figure 7:**
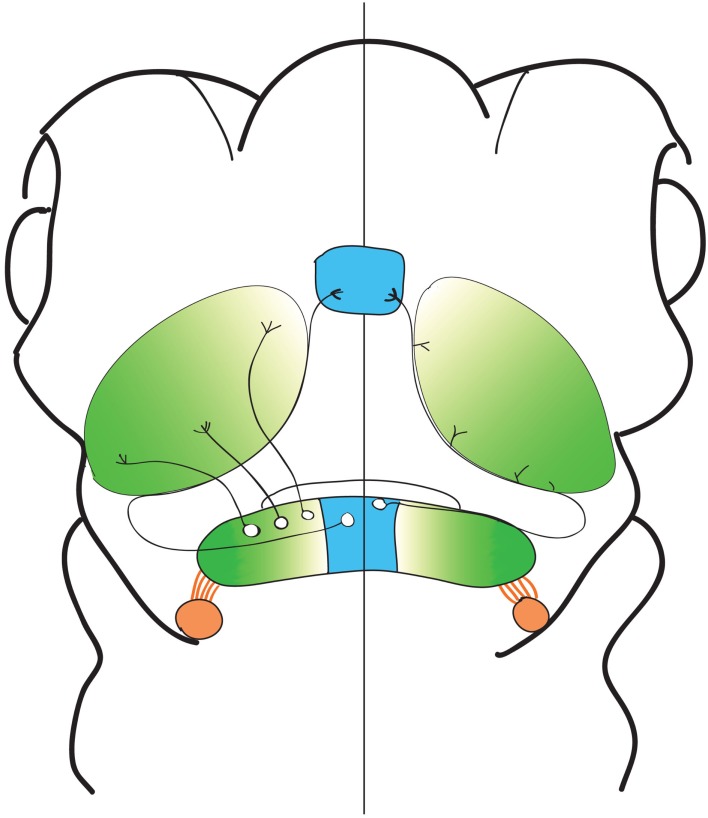
**Spatial patterns of eurydendroid projections**. A cartoon representation of the observed spatial organization of cerebellar output. Areas sending output to the thalamus, represented in blue, were confined to the most medial portions of the cerebellum, and areas sending output exclusively to the tectal neuropil were spread across the remainder of the medial-lateral axis. Topography was seen in the output to the optic tectum, with more medial cells projecting to rostral areas of the tectum, and more lateral areas projecting to the caudal areas of the cerebellum, represented by green shading. Axon tracts were visualized connecting the eminentia granularis to the CC (orange shading), but this was not observed at a single cell level.

In this study, we report the structural characteristics of eurydendroid cells, *en masse* and individually, in zebrafish larvae (Figure 7). We find that these neurons project principally to the tectal neuropil and the thalamus, and report a spatial logic in which the most medially positioned eurydendroid cells project axons through the tectal neuropil to the thalamus, while cells located throughout the remainder of the medial-lateral axis project exclusively to the tectal neuropil. Among these tectally projecting eurydendroid cells, we observe topography in which more medial eurydendroid cells extend axons to the rostral tectal neuropil while more lateral cells project to the caudal tectal neuropil. Eurydendroid axon terminals in the tectum are restricted to the deep layers of the neuropil, and since thalamus-bound eurydendroid axons also appear to synapse while passing through the tectal neuropil, these deep tectal targets receive a bulk of cerebellar output, at least among the cells represented in this study. Evidence supporting the existence of cerebellar output to the tectum has been previously observed in other teleost species including the scorpion fish (Murakami and Morita, 1987), long nose garfish (Northcutt, 1982), and catfish (Finger, 1983), although other studies have not observed these connections (Fiebig et al., 1983; Finger, 1983; Ikenaga et al., 2006). As well as the direct cerebello-tectal projections in teleost species, it has been shown that an indirect pathway between the valvula cerebellum and the optic tectum (via the torus semicircularis) exists (Folgueira et al., 2006, 2007). At a broad structural level, we did observe axons exiting the valvula cerebellum and terminating in the vicinity of the torus longitudinalis (Movie S1 in Supplementary Material), but these cells were not apparent in our single cell analysis, preventing us from comparing them to the ones described by Folgueira et al. (2006, 2007).

Surprisingly, projections to the hindbrain are faint in the *Gal4^s1168t^* expression pattern as a whole, and are not represented in our single cell analysis. Since this output has been described previously in mammals (Armstrong and Harvey, 1966; Sedgwick and Williams, 1967), and teleosts (Szabo, 1983; Murakami and Morita, 1987; Wullimann and Northcutt, 1988; Bae et al., 2009) we believe that their absence from this analysis results from proportionally small numbers, the possibility that they fail to express Gal4 in the *Gal4^s1168t^* line, or perhaps non-random labeling of cells using *BGUG* and variegated Brainbow. With this in mind, the results of this study should be viewed as a description of thalamic- and tectal-projecting eurydendroid cells rather than a comprehensive catalog of all cerebellar output in zebrafish larvae.

In adults of other teleost species, it has been shown that projections from the cerebellum are both contralateral and ipsilateral, with the majority being contralateral (Murakami and Morita, 1987; Ikenaga et al., 2006). In one case, the use of young adult fish indicated that the majority of eurydendroid cells projected ipsilaterally (Folgueira et al., 2006), and whether this is maintained in later-stage fish is uncertain. In contrast to this, all single cells that we have described project ipsilaterally. This raises the possibility that there is a gradual transition from ipsilateral cerebellar output early in development to predominantly contralateral output in adults. Other possibilities include sampling bias in our single cell labeling like that proposed above, or inter-species differences in the levels of midline crossing of cerebellar output axons.

### Eurydendroid diversity and regional specialization in the cerebellum

As described above, eurydendroid cells fill the same role in teleosts that the DCN do in mammals. Given the complexity of the DCN in terms of cell type diversity and connectivity, it is surprising that a single cell type could be sufficient for this role. In Japanese scorpion fish and goldfish, both teleosts, two subtypes of eurydendroid cells have been identified in adults that differ in terms of morphology, with either monopolar or multipolar morphologies (Murakami and Morita, 1987; Ikenaga et al., 2002). Efferent axons from monopolar eurydendroid cells are thought to project into the vicinity of the oculomotor complex, whereas multipolar cells project to all cerebellar targets (Murakami and Morita, 1987). Likewise, two categories of eurydendroid cells have been reported in zebrafish (Bae et al., 2009) and mormyrid fish (Meek et al., 1986a,b), based on the presence or absence of *calretinin* expression, although the anatomical and functional correlates of this expression are unclear.

Here, we show that there are at least two subtypes of eurydendroid cell in the larval zebrafish cerebellum, and it is likely that additional subtypes, projecting to the hindbrain and elsewhere, are missed by the current analysis. One subtype projects to the tectum only, consistently terminates in the very deepest layers of the tectal neuropil, and forms a topographic map between the cerebellum and the tectum. The other projects to the thalamus, crossing the tectal neuropil *en route*. Within the tectal neuropil, these axons course through the ventral and medial margin, extending short processes axially into the neuropil. Assuming that the tips of these processes contain pre-synaptic terminals, it appears likely that they innervate a slightly more superficial layer than the tectum-only eurydendroid cells do (data not shown), although the small number of cells that we have described in this category precludes a quantitative confirmation of this observation. Previously, it has been shown that cerebellar output to the thalamus passes through the medial longitudinal fascicle in adult goldfish (Ikenaga et al., 2002). In contrast to this, we see monosynaptic cerebellar outputs to the thalamus passing along the medial edge of the optic tectum. These differences may be due either to the differences in age, suggesting a remodeling between larval and adult fish, or to a difference between zebrafish and goldfish.

The two subtypes’ distinct targeting suggests that they may be playing different circuit roles, and therefore may be receiving distinct types of input. This possibility is supported by the spatial observation that medial eurydendroid cells project to the tectum and the thalamus while the remainder project only to the tectum. Characterization of the eurydendroid cell markers *calb2b* and *olig2* in the cerebellum has shown that they are expressed in a region specific manner, with medial (dorsal) eurydendroid cells expressing *olig2* and lateral (ventral) eurydendroid cells expressing *calb2b* (McFarland et al., 2008). This alignment of spatial characteristics between prior gene expression studies and our current anatomical work raises the possibility that our medial thalamic-projecting eurydendroid cells are the *olig2*-positive population and our more lateral tectal-projecting cells express *calb2b*. This suggests that regions of functional specialization across the cerebellar medial-lateral axis exist as is seen in mammals, where the DCN have distinct gene expression, physiology, connectivity, and behavioral relevance. A better understanding of these specialized regions in teleosts will await more detailed descriptions both of the inputs to different medial-lateral positions within the cerebellum and of the tectal and thalamic circuits into which these two types of eurydendroid cells synapse.

### Functional implications of eurydendroid anatomy

Projections from the cerebellum to the thalamus have been described in numerous systems, including goldfish (Ikenaga et al., 2002), rainbow trout (Folgueira et al., 2006), and mammals including rats, humans, and dogs (Person et al., 1986; Aumann et al., 1994; Gallay et al., 2008). Indeed, in mammals, the thalamus is one of the primary targets of the DCN, and the cerebello-thalamo-cortical pathway is critical to cerebellar function as a whole. It is perhaps surprising that a minority of our mapped eurydendroid cells project to the thalamus, and that these appear also to innervate the optic tectum. We cannot judge whether this results from a sampling bias in our experiments or a relative de-emphasis of cerebello-thalamic signaling in zebrafish. The latter, however, is plausible, given dramatic differences in connectivity between the thalamus and the telencephalon in teleosts versus mammals (reviewed by Mueller, 2012). Teleosts have telencephalic structures broadly homologous to the mammalian cerebral cortex (Northcutt, 2006), but the degree to which they possess structures homologous to the neocortex is debated (Wullimann and Mueller, 2004; Ito and Yamamoto, 2009). Because of the differences between the forebrain structures in mammals and teleosts, it is likely that the storage and tuning of motor programs in the two are carried out differently, with correspondingly different demands on communication among the cerebellum, thalamus, and telencephalon. It is also possible that thalamic output in mammals is proportionally heavier as a result of the mammalian expansion of the cerebellar hemispheres, which provide output to the cerebral cortex via the thalamus.

The optic tectum, in contrast, receives the exclusive output of a majority of the eurydendroid cells that we have observed, and may be post-synaptic even to those fibers that continue on to the thalamus. The robustness of this connection between the cerebellum and the tectum may speak to a major role for the tectum in relaying cerebellar output in teleosts. Within the tectal neuropil, eurydendroid terminals are seen exclusively in the deep layers. This is not surprising, since the superficial layers are primarily retinorecipient (Stuermer, 1988). Deep layers receive processed visual information from superficial tectal layers (Del Bene et al., 2010), mechanosensory information from the lateral line, as well as auditory and somatosensory inputs (Nederstigt and Schellart, 1986; Kinoshita et al., 2006), and they also generate the tectum’s output (Scott and Baier, 2009). These deep layers can therefore be viewed as a point of integration for several types of input, and as a result have the potential to produce output informed by a range of sensory and motor information (reviewed by Nevin et al., 2010). In larval zebrafish, output from the deep tectal layers goes to the superior raphe nucleus, the hindbrain reticular formation, the medulla oblongata, and possibly other targets (Sato et al., 2007; Scott and Baier, 2009). This puts the tectum in a position to blend sensory information with feedback from the cerebellum, and to relay this information to motor centers in the hindbrain.

The retino-tectal map is a textbook example of topography, where a retinal ganglion cell’s position in the retina determines its axon’s termination point in the tectum. The result is a spatial representation of the visual world in the tectum (or superior colliculus) that is conserved among vertebrates. In mammals, deep layers of the superior colliculus also have a topographic auditory map (Palmer and King, 1982), and these signals (along with visual signals from the retino-tectal map) contribute to the control of saccadic eye movements (Jay and Sparks, 1987a,b; Sparks and Hartwich-Young, 1989). Here, we report an additional topographic input to the tectum: that of the cerebellar eurydendroid cells. The implications of these overlaid topographic maps are not immediately obvious because the spatial underpinnings of tectal function are poorly understood in zebrafish (aside from retinal inputs). Since several of the modalities (auditory, visual, somatosensory, and proprioceptive) involved in motor calibration have a spatial component, it is possible that the tectum serves as a location for registering these modalities against one another spatially. Further detailed descriptions of tectal inputs will be necessary to see whether this is indeed the case.

### Limitations of this study, and suggestions for further research

As is always the case with purely anatomical studies, our results merely provide clues as to how these circuits function. Describing a circuit in terms of its cell type diversity, spatial arrangement, and connectivity is a prerequisite for understanding how activity through that circuit can drive behavior, but it is only a start. In the case of the teleost cerebellum, it will be interesting to identify the upstream and downstream neurons and circuits, and to see how output from the cerebellum is integrated with sensory and motor information from throughout the body. It will also be important to link these anatomical descriptions to function. This will be best approached by observing patterns of eurydendroid activity during behaviors that depend on the cerebellum, and by testing the impacts of eurydendroid silencing on the performance of these behaviors. Ever-improving optical tools for studying connectivity, and for observing and manipulating neural activity, dovetail with the transparency of zebrafish larvae. The concurrent development of new behavioral readouts for motor coordination (McClenahan et al., 2012) and learning (Aizenberg and Schuman, 2011; Ahrens et al., 2012) in zebrafish make this an appealing avenue for future research into the structure and function of cerebellar circuits.

## Conflict of Interest Statement

The authors declare that the research was conducted in the absence of any commercial or financial relationships that could be construed as a potential conflict of interest.

## Supplementary Material

The Supplementary Material for this article can be found online at http://www.frontiersin.org/Neural_Circuits/10.3389/fncir.2013.00053/abstract

Supplementary Movie S1 **A *z*-series of the Gal4s1168t; UAS:kaede line, in a 6 dpf larva**. Image is dorsal side up, with the anterior to the top of the image. Interval between slices is 3.94 μm. Scale bar represents 100 μm.

Supplementary Movie S2 **A *z*-series of the Gal4s1168t; UAS:kaede, UAS: syn-GFP line, 6 dpf, with photoconverted Kaede shown in red and synaptically targeted GFP shown as green**. Image is dorsal side up, with the anterior to the top of the image. Interval between slices is 1.60 μm. Scale bar represents 100 μm.

Supplementary Movies S3 and S4 **A *z*-series of a 6 dpf Gal4s1168t; UAS:kaede, UAS:BGUG larva with a pair of eurydendroid cells located in the medial cerebellum and projecting to the thalamus, and an intermediate eurydendroid cell projecting to the optic tectum**. Supplementary Movie 3: A *z*-series of the green channel (GFP) shown in Figures 4A′,B′. Supplementary Movie 4: A *z*-series of the merged green and red channels (GFP and photoconverted Kaede) shown in Figures 4A″,4B″. Interval between slices is 1.60 μm. Scale bar represents 100 μm.

## References

[B1] Ahrens B. Li J. M. Orger B. Robson N. Schier A. F. Engert F. (2012). Brain-wide neuronal dynamics during motor adaptation in zebrafish. Nature 485, 471–477 22622571 10.1038/nature11057 PMC3618960

[B2] Aizenberg M. Schuman E. M. (2011). Cerebellar-dependent learning in larval zebrafish. J. Neurosci. 31, 8708–8712 10.1523/JNEUROSCI.6565-10.2011 21677154 PMC6622926

[B3] Albus J. S. (1971). A theory of cerebellar function. Math. Biosci. 10, 25–61 10.1016/0025-5564(71)90051-4

[B4] Ando R. Hama H. Yamamoto-Hino M. Mizuno H. Miyawaki A. (2002). An optical marker based on the UV-induced green-to-red photoconversion of a fluorescent protein. Proc. Natl. Acad. Sci. U.S.A. 99, 12651–12656 10.1073/pnas.202320599 12271129 PMC130515

[B5] Andrezik J. A. Dormer K. J. Foreman R. D. Person R. J. (1984). Fastigial nucleus projections to the brain stem in beagles: pathways for autonomic regulation. Neuroscience 11, 497–507 10.1016/0306-4522(84)90040-X 6201783

[B6] Armstrong B. D. Harvey R. J. (1966). Responses in the inferior olive to stimulation of the cerebellar and cerebral cortices in the cat. J. Physiol. (Lond.) 187, 553–574 16783911 10.1113/jphysiol.1966.sp008108 PMC1395949

[B7] Asakawa K. Suster M. L. Mizusawa K. Nagayoshi S. Kotani T. Urasaki A. (2008). Genetic dissection of neural circuits by Tol2 transposon-mediated Gal4 gene and enhancer trapping in zebrafish. Proc. Natl. Acad. Sci. U.S.A. 105, 1255–1260 10.1073/pnas.0704963105 18202183 PMC2234125

[B8] Aumann T. D. Rawson J. A. Finkelstein D. I. Horne M. K. (1994). Projections from the lateral and interposed cerebellar nuclei to the thalamus of the rat: a light and electron microscopic study using single and double anterograde labelling. J. Comp. Neurol. 349, 165–181 10.1002/cne.903490202 7860776

[B9] Bae Y. K. Kani S. Shimizu T. Tanabe K. Nojima H. Kimura Y. (2009). Anatomy of zebrafish cerebellum and screen for mutations affecting its development. Dev. Biol. 330, 406–426 10.1016/j.ydbio.2009.04.013 19371731

[B10] Bass A. H. (1982). Evolution of the vestibulolateral lobe of the cerebellum in electroreceptive and nonelectroreceptive teleosts. J. Morphol. 174, 335–348 10.1002/jmor.1051740306 30086606

[B11] Becanovic K. Pouladi M. A. Lim R. S. Kuhn A. Pavlidis P. Luthi-Carter R. (2010). Transcriptional changes in Huntington disease identified using genome-wide expression profiling and cross-platform analysis. Hum. Mol. Genet. 19, 1438–1452 10.1093/hmg/ddq018 20089533 PMC2846159

[B12] Blakemore S. J. Wolpert D. M. Frith C. D. (1998). Central cancellation of self-produced tickle sensation. Nat. Neurosci. 1, 635–640 10.1038/2870 10196573

[B13] Brand A. H. Perrimon N. (1993). Targeted gene expression as a means of altering cell fates and generating dominant phenotypes. Development 118, 401–415 8223268 10.1242/dev.118.2.401

[B14] Carey M. R. (2011). Synaptic mechanisms of sensorimotor learning in the cerebellum. Curr. Opin. Neurobiol. 21, 609–615 10.1016/j.conb.2011.06.011 21767944

[B15] Collin S. P. Pettigrew J. D. (1988). Retinal topography in reef teleosts. I. Some species with well-developed areae but poorly-developed streaks. Brain Behav. Evol. 31, 269–282 10.1159/000116595 3395836

[B16] Davison J. M. Akitake C. M. Goll M. G. Rhee J. M. Gosse N. Baier H. (2007). Transactivation from Gal4-VP16 transgenic insertions for tissue-specific cell labeling and ablation in zebrafish. Dev. Biol. 304, 811–824 10.1016/j.ydbio.2007.01.033 17335798 PMC3470427

[B17] Del Bene F. Wyart C. Robles E. Tran A. Looger L. Scott E. K. (2010). Filtering of visual information in the tectum by an identified neural circuit. Science 330, 669–673 10.1126/science.1192949 21030657 PMC3243732

[B18] Distel M. Wullimann M. F. Koster R. W. (2009). Optimized Gal4 genetics for permanent gene expression mapping in zebrafish. Proc. Natl. Acad. Sci. U.S.A. 106, 13365–13370 10.1073/pnas.0903060106 19628697 PMC2726396

[B19] Donarum E. A. Stephan D. A. Larkin K. Murphy E. J. Gupta M. Senephansiri H. (2006). Expression profiling reveals multiple myelin alterations in murine succinate semialdehyde dehydrogenase deficiency. J. Inherit. Metab. Dis. 29, 143–156 10.1007/s10545-006-0247-6 16601881

[B20] Dow R. S. Moruzzi G. (1958). The Physiology and Pathology of the Cerebellum. Minneapolis: The University of Minnesota Press

[B21] Eccles J. D. (1971). Functional significance of arrangement of neurones in cell assemblies. Arch. Psychiatr. Nevenkr. 215, 92–106 10.1007/BF00342825 4333691

[B22] Emmenlauer M. Ronneberger O. Ponti A. Schwarb P. Griffa A. Filippi A. (2009). XuvTools: free, fast and reliable stitching of large 3D datasets. J. Microsc. 233, 42–60 10.1111/j.1365-2818.2008.03094.x 19196411

[B23] Feng G. Mellor R. H. Bernstein M. Keller-Peck C. Nguyen Q. T. Wallace M. (2000). Imaging neuronal subsets in transgenic mice expressing multiple spectral variants of GFP. Neuron 28, 41–51 10.1016/S0896-6273(00)00145-8 11086982

[B24] Fiebig E. Ebbesson S. O. E. Meyerm D. L. (1983). Afferent connections of the optic tectum in the piranha (*Serrasalmus nattereri*). Cell Tissue Res. 231, 55–72 10.1007/BF00215774 6850798

[B25] Finger T. E. (1983). Organization of the teleost cerebellum. Fish Neurobiol. 1, 261–284

[B26] Folgueira M. Anadon R. Yanez J. (2006). Afferent and efferent connections of the cerebellum of a salmonid, the rainbow trout (*Oncorhynchus mykiss*): a tract-tracing study. J. Comp. Neurol. 497, 542–565 10.1002/cne.20979 16739164

[B27] Folgueira M. Sueiro C. Rodriguez-Moldes I. Yanez J. Anadon R. (2007). Organization of the torus longitudinalis in the rainbow trout (*Oncorhynchus mykiss*): an immunohistochemical study of the GABAergic system and a DiI tract-tracing study. J. Comp. Neurol. 503, 348–370 10.1002/cne.21363 17492628

[B28] Friedrich R. W. Jacobson G. A. Zhu P. (2010). Circuit neuroscience in zebrafish. Curr. Biol. 20, R371–R381 10.1016/j.cub.2010.02.039 21749961

[B29] Gallay M. N. Jeanmonod D. Liu J. Morel A. (2008). Human pallidothalamic and cerebellothalamic tracts: anatomical basis for functional stereotactic neurosurgery. Brain Struct. Funct. 212, 443–463 10.1007/s00429-007-0170-0 18193279 PMC2494572

[B30] Glickstein M. Sultan F. Voogd J. (2011). Functional localization in the cerebellum. Cortex 47, 59–80 10.1016/j.cortex.2009.09.001 19833328

[B31] Hepp K. Henn V. Jaeger J. (1982). Eye movement related neurons in the cerebellar nuclei of the alert monkey. Exp. Brain Res. 45, 253–264 10.1007/BF00235785 7056331

[B32] Hibi M. Shimizu T. (2012). Development of the cerebellum and cerebellar neural circuits. Dev. Neurobiol. 72, 282–301 10.1002/dneu.20875 21309081

[B33] Hoebeek F. E. Witter L. Ruigrok T. J. De Zeeuw C. I. (2010). Differential olivo-cerebellar cortical control of rebound activity in the cerebellar nuclei. Proc. Natl. Acad. Sci. U.S.A. 107, 8410–8415 10.1073/pnas.0907118107 20395550 PMC2889566

[B34] Ikenaga T. Yoshida M. Uematsu K. (2002). Efferent connections of the cerebellum of the goldfish, *Carassius auratus*. Brain Behav. Evol. 60, 36–51 10.1159/000064120 12239469

[B35] Ikenaga T. Yoshida M. Uematsu K. (2006). Cerebellar efferent neurons in teleost fish. Cerebellum 5, 268–274 10.1080/14734220600930588 17134989

[B36] Ito H. Yamamoto N. (2009). Non-laminar cerebral cortex in teleost fishes? Biol. Lett. 5, 117–121 10.1098/rsbl.2008.0397 18832057 PMC2657732

[B37] Jay M. F. Sparks D. L. (1987a). Sensorimotor integration in the primate superior colliculus. I. Motor convergence. J. Neurophysiol. 57, 22–34 3559673 10.1152/jn.1987.57.1.22

[B38] Jay M. F. Sparks D. L. (1987b). Sensorimotor integration in the primate superior colliculus. II. Coordinates of auditory signals. J. Neurophysiol. 57, 35–55 3559680 10.1152/jn.1987.57.1.35

[B39] Kani S. Bae Y. K. Shimizu T. Tanabe K. Satou C. Parsons M. J. (2010). Proneural gene-linked neurogenesis in zebrafish cerebellum. Dev. Biol. 343, 1–17 10.1016/j.ydbio.2010.03.024 20388506

[B40] Kawakami K. (2004). Transgenesis and gene trap methods in zebrafish by using the Tol2 transposable element. Methods Cell Biol. 77, 201–222 10.1016/S0091-679X(04)77011-9 15602913

[B41] Kinoshita M. Ito E. Urano A. Ito H. Yamamoto N. (2006). Periventricular efferent neurons in the optic tectum of rainbow trout. J. Comp. Neurol. 499, 546–564 10.1002/cne.21080 17029270

[B42] Kotani T. Nagayoshi S. Urasaki A. Kawakami K. (2006). Transposon-mediated gene trapping in zebrafish. Methods 39, 199–206 10.1016/j.ymeth.2005.12.006 16814563

[B43] Kwan K. M. Fujimoto E. Grabher C. Mangum B. D. Hardy M. E. Campbell D. S. (2007). The Tol2kit: a multisite gateway-based construction kit for Tol2 transposon transgenesis constructs. Dev. Dyn. 236, 3088–3099 10.1002/dvdy.21343 17937395

[B44] Laplante M. Kikuta H. König M. Becker T. S. (2006). Enhancer detection in the zebrafish using pseudotyped murine retroviruses. Methods 39, 189–198 10.1016/j.ymeth.2006.01.003 16887366

[B45] Li L. Tasic B. Micheva K. D. Ivanov V. M. Spletter M. L. Smith S. J. (2010). Visualizing the distribution of synapses from individual neurons in the mouse brain. PLoS ONE 5:e11503 10.1371/journal.pone.0011503 20634890 PMC2901335

[B46] Lister J. A. Robertson C. P. Lepage T. Johnson S. L. Raible D. W. (1999). Nacre encodes a zebrafish microphthalmia-related protein that regulates neural-crest-derived pigment cell fate. Development 126, 3757–3767 10433906 10.1242/dev.126.17.3757

[B47] Livet J. Weissman T. A. Kang H. Draft R. W. Lu J. Bennis R. A. (2007). Transgenic strategies for combinatorial expression of fluorescent proteins in the nervous system. Nature 450, 56–62 10.1038/nature06293 17972876

[B48] Longair M. H. Baker D. A. Armstrong J. D. (2011). Simple neurite tracer: open source software for reconstruction, visualization and analysis of neuronal processes. Bioinformatics 27, 2453–2454 10.1093/bioinformatics/btr390 21727141

[B49] Luo L. Callaway E. M. Svoboda K. (2008). Genetic dissection of neural circuits. Neuron 57, 634–660 10.1016/j.neuron.2008.01.002 18341986 PMC2628815

[B50] McClenahan P. Troup M. Scott E. K. (2012). Fin-tail coordination during escape and predatory behavior in larval zebrafish. PLoS ONE 7:e32295 10.1371/journal.pone.0032295 22359680 PMC3281131

[B51] McFarland K. A. Topczewska J. M. Weidinger G. Dorsky R. I. Appel B. (2008). Hh and Wnt signaling regulate formation of olig2+ neurons in the zebrafish cerebellum. Dev. Biol. 318, 162–171 10.1016/j.ydbio.2008.03.016 18423594 PMC2474464

[B52] Meek J. Nieuwenhuys R. Elsavier D. (1986a). Afferent and efferent connections of cerebellar lobe C_1_ of the mormyrid fish *Gnathonemus petersi*: an HRP study. J. Comp. Neurol. 245, 319–341 10.1002/cne.902450304 3958249

[B53] Meek J. Nieuwenhuys R. Elsevier D. (1986b). Afferent and efferent connections of cerebellar lobe C3 of the mormyrid fish *Gnathonemus petersi*: an HRP study. J. Comp. Neurol. 245, 342–358 10.1002/cne.902450304 2870092

[B54] Meyer M. P. Smith S. J. (2006). Evidence from in vivo imaging that synaptogenesis guides the growth and branching of axonal arbors by two distinct mechanisms. J. Neurosci. 26, 3604–3614 10.1523/JNEUROSCI.0223-06.2006 16571769 PMC6673851

[B55] Miall R. C. Weir D. J. Wolpert D. M. Stein J. F. (1993). Is the cerebellum a smith predictor? J. Mot. Behav. 25, 203–216 10.1080/00222895.1993.9941639 12581990

[B56] Miles F. A. Lisberger S. G. (1981). Plasticity in the vestibulo-ocular reflex: a new hypothesis. Annu. Rev. Neurosci. 4, 273–299 10.1146/annurev.ne.04.030181.001421 6784658

[B57] Miyamura Y. Nakayasu H. (2001). Zonal distribution of Purkinje cells in the zebrafish cerebellum: analysis by means of a specific monoclonal antibody. Cell Tissue Res. 305, 299–305 10.1007/s004410100421 11572083

[B58] Mueller T. (2012). What is the thalamus in zebrafish? Front. Neurosci. 6:64 10.3389/fnins.2012.00064 22586363 PMC3345571

[B59] Murakami T. Morita Y. (1987). Morphology and distribution of the projection neurons in the cerebellum in a teleost, *Sebastiscus marmoratus*. J. Comp. Neurol. 256, 607–623 10.1002/cne.902560413 3558892

[B60] Nederstigt L. J. Schellart N. A. (1986). Acousticolateral processing in the torus semicircularis of the trout *Salmo gairdneri*. Pflugers Arch. 406, 151–157 10.1007/BF00586676 3960698

[B61] Nevin L. M. Robles E. Baier H. Scott E. K. (2010). Focusing on optic tectum circuitry through the lens of genetics. BMC Biol. 8:126 10.1186/1741-7007-8-126 20920150 PMC2949621

[B62] Niell C. M. Meyer M. P. Smith S. J. (2004). In vivo imaging of synapse formation on a growing dendritic arbor. Nat. Neurosci. 7, 254–260 10.1038/nn1191 14758365

[B63] Northcutt R. G. (1982). Localization of neurons afferent to the optic tectum in longnose gars. J. Comp. Neurol. 204, 325–335 10.1002/cne.902040404 7061736

[B64] Northcutt R. G. (2006). Connections of the lateral and medial divisions of the goldfish telencephalic pallium. J. Comp. Neurol. 494, 903–943 10.1002/cne.20853 16385483

[B65] Palmer A. R. King A. J. (1982). The representation of auditory space in the mammalian superior colliculus. Nature 299, 248–249 10.1038/299248a0 7110344

[B66] Person R. J. Andrezik J. A. Dormer K. J. Foreman R. D. (1986). Fastigial nucleus projections in the midbrain and thalamus in dogs. Neuroscience 18, 105–120 10.1016/0306-4522(86)90182-X 2426627

[B67] Sajovic P. Levinthal C. (1982). Visual cells of zebrafish optic tectum: mapping with small spots. Neuroscience 7, 2407–2426 10.1016/0306-4522(82)90204-4 7177381

[B68] Sato T. Hamaoka T. Aizawa H. Hosoya T. Okamoto H. (2007). Genetic single-cell mosaic analysis implicates ephrinB2 reverse signaling in projections from the posterior tectum to the hindbrain in zebrafish. J. Neurosci. 27, 5271–5279 10.1523/JNEUROSCI.4218-06.2007 17507550 PMC6672335

[B69] Scheer N. Campos-Ortega J. A. (1999). Use of the Gal4-UAS technique for targeted gene expression in the zebrafish. Mech. Dev. 80, 153–158 10.1016/S0925-4773(98)00209-3 10072782

[B70] Scott E. K. (2009). The Gal4/UAS toolbox in zebrafish: new approaches for defining behavioral circuits. J. Neurochem. 110, 441–456 10.1111/j.1471-4159.2009.06161.x 19457087

[B71] Scott E. K. Baier H. (2009). The cellular architecture of the larval zebrafish tectum, as revealed by gal4 enhancer trap lines. Front. Neural Circuits 3:13 10.3389/neuro.04.013.2009 19862330 PMC2763897

[B72] Scott E. K. Mason L. Arrenberg A. B. Ziv L. Gosse N. J. Xiao T. (2007). Targeting neural circuitry in zebrafish using GAL4 enhancer trapping. Nat. Methods 4, 323–326 17369834 10.1038/nmeth1033

[B73] Sedgwick E. M. Williams T. D. (1967). Responses of single units in the inferior olive to stimulation of the limb nerves, peripheral skin receptors, cerebellum, caudate nucleus and motor cortex. J. Physiol. (Lond.) 189, 261–279 5340538 10.1113/jphysiol.1967.sp008167 PMC1396060

[B74] Simmich J. Staykov E. Scott E. (2012). Zebrafish as an appealing model for optogenetic studies. Prog. Brain Res. 196, 145–162 10.1016/B978-0-444-59426-6.00008-2 22341325

[B75] Simpson H. D. Kita E. M. Scott E. K. Goodhill G. J. (2013). A quantitative analysis of branching, growth cone turning and directed growth in zebrafish retinotectal axon guidance. J. Comp. Neurol. 521, 1409–1429 10.1002/cne.23248 23124714

[B76] Sparks D. L. Hartwich-Young R. (1989). The deep layers of the superior colliculus. Rev. Oculomot. Res. 3, 213–255 2486324

[B77] Stuermer C. A. (1988). Retinotopic organization of the developing retinotectal projection in the zebrafish embryo. J. Neurosci. 8, 4513–4530 2848935 10.1523/JNEUROSCI.08-12-04513.1988 PMC6569580

[B78] Sultan F. Augath M. Hamodeh S. Murayama Y. Oeltermann A. Rauch A. (2012). Unravelling cerebellar pathways with high temporal precision targeting motor and extensive sensory and parietal networks. Nat. Commun. 3, 924 10.1038/ncomms1912 22735452

[B79] Szabo T. (1983). Cerebellar pathways in the brain of the mormyrid teleost fish. Acta Morphol. Hung. 31, 219–234 6414258

[B80] Thach W. T. (1968). Discharge of Purkinje and cerebellar nuclear neurons during rapidly alternating arm movements in the monkey. J. Neurophysiol. 31, 785–797 4974877 10.1152/jn.1968.31.5.785

[B81] Thierry-Mieg D. Thierry-Mieg J. (2006). AceView: a comprehensive cDNA-supported gene and transcripts annotation. Genome Biol. 7(Suppl. 1–12), 1–14 10.1186/gb-2006-7-1-r1 16925834 PMC1810549

[B82] Tseng Y. W. Diedrichsen J. Krakauer J. W. Shadmehr R. Bastian A. J. (2007). Sensory prediction errors drive cerebellum-dependent adaptation of reaching. J. Neurophysiol. 98, 54–62 10.1152/jn.00266.2007 17507504

[B83] Westerfield M. (2000). The Zebrafish Book. A Guide for the Laboratory Use of Zebrafish (Danio rerio), 4th Edn Eugene: University of Oregon Press

[B84] Wullimann M. F. Mueller T. (2004). Teleostean and mammalian forebrains contrasted: evidence from genes to behavior. J. Comp. Neurol. 475, 143–162 10.1002/cne.20183 15211457

[B85] Wullimann M. F. Northcutt R. G. (1988). Connections of the corpus cerebelli in the green sunfish and the common goldfish: a comparison of perciform and cypriniform teleosts. Brain Behav. Evol. 32, 293–316 10.1159/000116558 3233488

[B86] Wyart C. Del Bene F. Warp E. Scott E. K. Trauner D. Baier H. (2009). Optogenetic dissection of a behavioural module in the vertebrate spinal cord. Nature 461, 407–410 10.1038/nature08323 19759620 PMC2770190

[B87] Xiao T. Roeser T. Staub W. Baier H. (2005). A GFP-based genetic screen reveals mutations that disrupt the architecture of the zebrafish retinotectal projection. Development 132, 2955–2967 10.1242/dev.01861 15930106

[B88] Zapala M. A. Hovatta I. Ellison J. A. Wodicka L. Del Rio J. A. Tennant R. (2005). Adult mouse brain gene expression patterns bear an embryologic imprint. Proc. Natl. Acad. Sci. U.S.A. 102, 10357–10362 10.1073/pnas.0503357102 16002470 PMC1173363

[B89] Zhang Y. Magnus G. Han V. Z. (2008). Local circuitry in the anterior lateral caudal lobe of the mormyrid cerebellum: a study of intracellular recording and labelling. J. Comp. Neurol. 509, 1–22 10.1002/cne.21750 18418897

